# Communication in COVID: a quality improvement project into staff communication with family/carers at New Haven Older Adult Mental Health Inpatient Unit

**DOI:** 10.1192/bjo.2021.534

**Published:** 2021-06-18

**Authors:** Felicity Jones, Bhavna Khanna, Batool Almoosawi, Alex Humm, Upjeet Mahon, Rosie Edwards

**Affiliations:** 1 Hereford and Worcestershire Health and Care NHS Trust; 2 University of Birmingham, Medical School

## Abstract

**Aims:**

In the psychiatric care of patients, family involvement is key to recovery. At the New Haven Unit, there have been a number of complaints regarding poor communication and lack of updates given to families during COVID-19.

The aim is to:

To increase the overall satisfaction of the family with the service received for their loved ones

Ensure effective and timely communication of updates to the families, to prevent further complaints, by assigning a member of staff per patient to be the primary individual responsible for family contact

Create an addition to the weekly ward round MDT proforma on ‘Carenotes’ where communication can be documented

**Method:**

A standardised questionnaire has been sent to the relatives of inpatients at the New Haven Unit. Qualitative data are being collated, which will lead to quantitative statistical analysis of the satisfaction ratings.

Based on the current bed state on the ward at the time of the project all 32 relatives of current inpatients were contacted and 23 agreed to complete the survey which was sent out either by email or post.

The new MDT proforma will be added, which will be used to record actions needed to be taken involving communication and updating family members on a weekly basis. This opportunity to record communication will improve continuity of care and satisfaction amongst family members.

There will be follow-up via a second questionnaire to identify improvement.

**Result:**

The average results of selected categories so far are shown below (still awaiting further responses):

Frequency of updates regarding loved ones = 4.33/10 (10-excellent)

To what degree were your concerns listened to? 7.33/10

Quality of content discussed with staff members = 3.33/4 (4- excellent)

Other categories scoring below the expected standard, included awareness of visiting guidelines and questions regarding lasting power of attorney, in which 33.3% of participants responded either ‘no’ or ‘not sure’ respectively.

Questions addressing formalities of introduction and confidentiality through identity confirmation, scored highly.

**Conclusion:**

We are awaiting more survey responses in order to identify additional areas of improvement; however, it is already clear to see that there are areas that would be advanced through structured, assigned reminders via an MDT amendment.

We will also be introducing set dates for conference calls with the families now involving the whole MDT; one within the first week of admission, one after six weeks and one at the point of discharge as a minimum.

